# Surgical management and long-term follow-up of ocular hypotony associated with peripheral circumferential retinal detachment

**DOI:** 10.1186/s40942-025-00648-7

**Published:** 2025-03-10

**Authors:** Leandro Cabral Zacharias, Lívia da Silva Conci, Davi Paraguassu de Sousa Martins, Rony Carlos Preti, Leonardo Proveti Cunha, Mario Luiz Ribeiro Monteiro

**Affiliations:** 1 Department of Ophthalmology, University of Sao Paulo, Sao Paulo, Brazil; 2 Department of Ophthalmology, Federal University of Juiz de Fora, Minas Gerais, Brazil

**Keywords:** Ocular hypotension, Retinal detachment, Vitrectomy, Ciliary body

## Abstract

**Purpose:**

This study aimed to evaluate the surgical management and long-term follow-up of hypotony related to post-vitrectomy peripheral circumferential retinal detachment (PCD).

**Methods:**

Eyes diagnosed with hypotony secondary to PCD after pars plana vitrectomy were retrospectively reviewed. The patient demographic data, complications, management, and treatment outcomes were collected and analyzed.

**Results:**

Four eyes were included in this study. The median preoperative best-corrected visual acuity (BCVA) was 20/200 and the mean preoperative intraocular pressure (IOP) was 4.5 mmHg. One case had rubeosis iridis. All eyes underwent peripheral retinectomy to remove the detached retina and release ciliary body traction. Complete retinal reattachment was achieved in all eyes. The final BCVA ranged from 20/200 to 20/50 and regression of rubeosis iridis was observed. Mean IOP improved to 12,25 mmHg at 12 months after the retinectomy. The mean follow- up time was 5 years (range 2–7 years).

**Conclusion:**

Aggressive dissection and 360º trimming of the anterior retina is helpful to relieve ciliary body traction and remove ischemic tissue, restoring minimally adequate aqueous production in cases of hypotony related to PCD.

**Trial registration:**

This retrospective study was approved by the Institutional Review Board, under registration number 77599724.7.0000.0068.

**Supplementary Information:**

The online version contains supplementary material available at 10.1186/s40942-025-00648-7.

## Background

Although infrequent, ocular hypotony after rhegmatogenous retinal detachment repair is a potentially vision-threatening condition [1]. According to statistical criteria, hypotony is defined as intraocular pressure (IOP) less than 6.5 mmHg (3 standard deviations below the mean) [2]. Ocular hypotony results from a disturb in aqueous humor dynamics and may be a complication in operated eyes, including those submitted to posterior pars plana vitrectomy [1]. 

Proliferative vitreoretinopathy (PVR) is the most common cause for failure of rhegmatogenous retinal detachment (RRD) repair. It is characterized by proliferative, contractile cellular membranes formation in the vitreous and on both sides of the retina, as well as intraretinal contraction [3]. There is a specific form of recurrent RRD, known as peripheral circumferential detachment (PCD), in which retinal detachment is located circumferentially at the peripheral area (anterior to the equator), and restricted to it usually by chorioretinal adhesions [4], in a conformation referred in literature as “donut shapedretinal detachment”. In these cases, anterior PVR can occur, causing ciliary body traction and chronic hypotony [5]. This can lead to poor vision and phthisis development, despite most of the retina anatomically reattached [5].

In the present study, we evaluated the surgical management and long-term follow-up of hypotony related to post-vitrectomy PCD. We also described the employed surgical technique, without the use of endoscopy.

## Methods

This retrospective case series study was conducted at the University of Sao Paulo Medical School Clinics Hospital (HCFMUSP), located in Sao Paulo-SP, Brazil. It was approved by the Institutional Review Board, under registration number 77599724.7.0000.0068.

The inclusion criteria were (1) recurrent RD after vitrectomy showing the features of PCD at fundus examination and (2) development of hypotony (IOP < 6.5 mmHg). PCD is defined as a recurrent RD limited to the periphery in a circumferential fashion. It is limited to the periphery by equatorial laser-induced chorioretinal scars [4]. PCD was diagnosed mainly based on the findings of clinical examination.

Medical records of the included cases were analyzed. Data regarding age, sex, previous surgical procedures, rubeosis iridis, lens status, operation findings, complications, and follow-up duration, were revised. Ophthalmic examinations, including best-corrected visual acuity (BCVA) measurements, IOP measurements, indirect ophthalmoscopy, and color fundus photography were compiled.

## Results

### Patient and peripheral circumferential retinal detachment characteristics

There were four eyes of PCD in four patients included in this study, presenting between 2013 and 2018. Patient ages ranged from 28 to 54 years. There was no gender predominance (male/female: 1:1). All cases were refered to our service after one or more surgical interventions in other centers.

The median preoperative visual acuity was 20/200. The mean follow-up time was 5 years (range 2–7 years). The mean preoperative IOP was 4.5 mmHg, and one case had rubeosis iridis. All patients had at least one prior surgery for RRD (pars plana vitrectomy). Previous diagnosis included rhegmatogenous retinal detachment (RRD) in three cases, and tractional retinal detachment (TRD) secondary to Eales’ disease in one case. Ultrasound biomicroscopy images were not available.

### Surgical technique

**Fig. 1:**
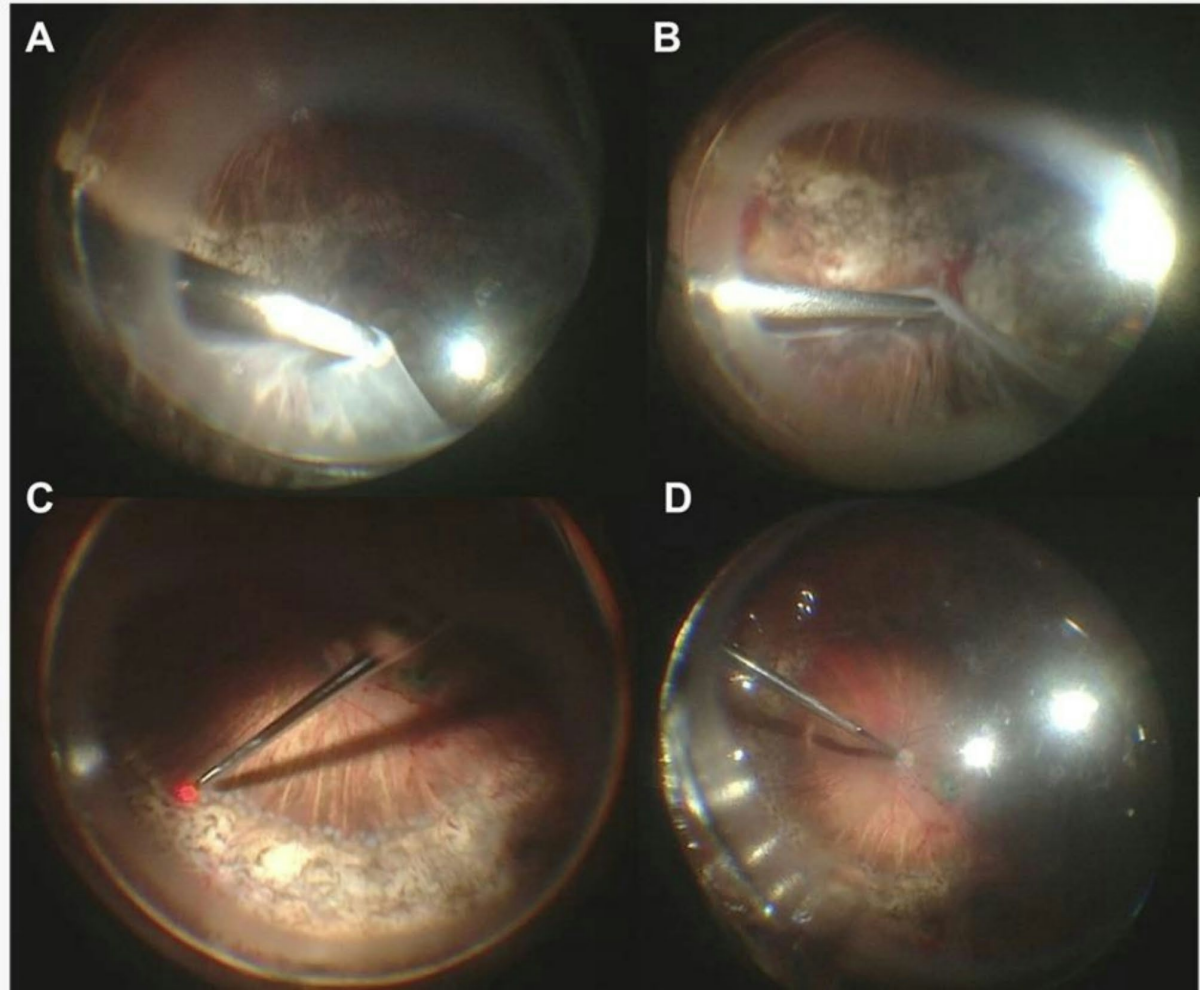
Intraoperative photographs of the surgical steps. (**A**) 360° trimming of the peripheral retina using the vitrectomy probe; (**B**) Aggressive dissection of the nonfunctional anterior retina up to the ciliary body; (**C**) Encircling rows of laser photocoagulation; (**D**) Fluid-air exchange and endotamponade with perfluoropropane (C3F8)

Before creating a retinotomy, meticulous diathermy was applied to the margin of the retina that was to be cut and perfluorocarbon was used to stabilize the center of the retina. After that, peripheral retina was trimmed using the vitrectomy probe. Special measures were taken to prevent choroidal and retinal hemorrhage while cutting the retina, such as dissection of shallow space with vitrectomy scissors or vitrectomy probe. All retinotomies were created as peripheral as possible. The retinotomy was followed by the aggressive trimming of the nonfunctional anterior retina up to the ciliary body. After the 360° retinectomy, encircling rows of laser photocoagulation were applied along the rim of the remaining posterior retina as needed, depending on previous surgeries laser scars placement. Perfluoropropane (C3F8) was used for endotamponade in all eyes. Intraoperative photographs are presented in Fig. 1. An example (case 2) is shown in Supplemental Video 1.

### Outcomes

All surgeries were performed by the same surgeon (LCZ) and all cases were successful. The pre and late post operative (at 12 months) visual acuity are presented in Table 1. Visual improvement occurred in three eyes at the twelfth month review and remained the same in one eye (case no. 4). There were no intraoperative complications.

The mean preoperative intraocular pressure was 4,5 mmHg. The meanpostoperative intraocular pressure was 12,25 mmHg at 12 months after the retinectomy. Regression of rubeosis iridis was observed in one of the patients (case no. 2).

**Fig. 2:**
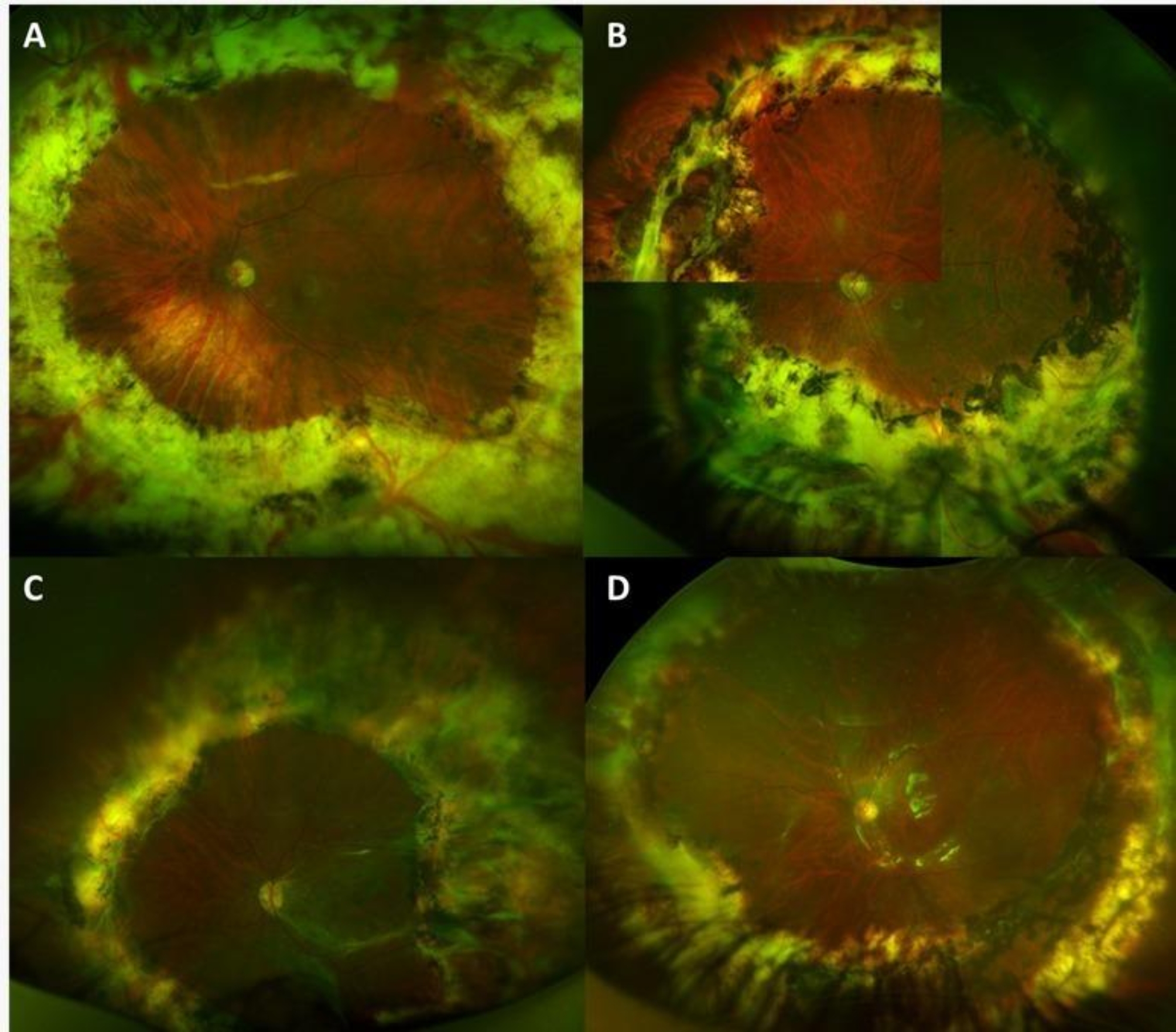
Late-Postoperative wide field fundus photography shows attached retina and 360° retinectomy in the four cases (**A**-**D**: cases 1 to 4, respectively)

The characteristics of the four cases are summarized in the Table 1. The final appearance after extensive retinectomy was documented by ultra-wide field fundus imaging and it is represented in Fig. 2.

**Table 1:** Summary of cases’ data

Case	Sex	Age (years)	Eye	Pre-IOP (mmHg)	Pre-BCVA	Iris rubeosis	Post-IOP (mmHg)	Post-BCVA	Diagnosis	Years of Follow-up
1	F	49	OS	6	20/200	no	11	20/50	RRD	7
2	M	54	OS	5	20/400	yes	16	20/200	RRD	6
3	F	43	OS	2	20/200	no	8	20/70	TRD	5
4	M	28	OS	5	20/200	no	14	20/200	RRD	2

## Discussion

This study reports the development of chronic hypotony associated with post-vitrectomy PCD in four patients. The hypotony after vitreous surgery can be explained by inadequate aqueous humor production secondary to tractional ciliary detachment related to anterior PVR [6]. The mechanical separation of ciliary body from its natural position may result in disrupted nonpigmented ciliary epithelium and its vasculature, leading to a reduced aqueous production [1]. 

It has already been demonstrated that early surgery to release traction over the anterior retina and uveal tissue in eyes with chronic hypotony and anterior PVR can increase intraocular pressure and stabilize visual acuity [7, 8]. In our case series, all patients underwent successful revision surgery to remove the detached retina and release ciliary body traction. IOP level increase was achieved in all cases, with no reported complications.

It is worthy to consider that, in cases with large relaxing retinotomies, hypotony may be attributed to the exposed retinal pigment epithelium and increase of the uveoscleral outflow, further lowering the IOP [9]. However, if the anterior retinal flap is not excised, it can retract anteriorly, pulling on the pars plicata and causing hypotony [9]. In our series, the role of extensive retinectomy was precisely to relieve traction and increase IOP level. Despite extensive, the retinectomy was placed as peripheral as possible in order to minimize the increase in uveoscleral outflow.

Iris neovascularization was found just in one case. This finding was less frequent than in other studies [4, 10, 11]. Devitalized detached retina is an uncommon but already well stablished cause of neovascular glaucoma, as the ischemic retina may produce cytokines such as VEGF [10]. Anterior PVR may also be associated with vascular proliferation. It has already been demonstrated that hypotony occurs more commonly than neovascular glaucoma in eyes with PCD and rubeosis iridis, probably due to the low aqueous production, but it may lead to structural anterior chamber changes that can jeopardize eyeball stability. On the other hand, if aqueous outflow is blocked sufficiently by the new vessels, neovascular glaucoma may develop [12].

In our study, all surgeries successfully led to an increase in late postoperative IOP. Visual improvement occurred in three eyes after the operation and remained unchanged in one eye. Significant visual gain was observed in cases 1 and 3. This may be secondary to the resolution of a possible hypotonic maculopathy, following the increase in intraocular pressure levels after 360° retinectomy. However, the predictability of functional outcomes remains poor because of the wide range in interindividual postoperative visual acuity. We assume that visual acuity improvement was hampered by previous retinal conditions.

Regarding surgical approach, it is well-known that endoscopy-assisted pars plana vitrectomy (E-PPV) has some technical advantages over our surgical approach. It can be used to manage ciliary body pathologies by removing the epiciliary membranes, such as cyclitic membranes causing ciliary detachment and hypotony [13]. Therefore, E-PPV allows a complete examination and extensive anterior PVR and vitreous base dissection without scleral indentation [14]. However, few vitreoretinal surgeons have experience with E-PPV. Nevertheless, the surgical technique presented in this article can be safely performed with tools that are available in most vitreoretinal surgery centers. By contrast with other case series that used silicon oil [4, 11], C3F8 was chosen as endotamponade in all eyes. We believe silicone oil tamponade was not needed, as posterior pole was already attached and the RD was restricted to the area anterior to the equator.

There are some limitations in our study. First, selection bias may be present due to its retrospective nature and its monocentric setting. Besides, as hypotony related to PCD is a rare ocular condition, there is a limited number of evaluated eyes.

To prevent PCD and its complications, a meticulous surgical technique in the primary PPV is needed. Good practices, such as removing the peripheral vitreous, supporting the vitreous base, and sealing breaks, must be employed. However, in cases with PCD and hypotony, this study suggests that a 360° peripheral retinectomy for retinal-ciliary body traction release and aggressive distal retina trimming may be a valuable option to reverse hypotony and then preserve visual function and globe anatomy.

## Conclusion

In summary, our case series suggests that aggressive dissection and 360º trimming of the anterior retina is helpful to relieve ciliary body traction and remove ischemic tissue, restoring minimally adequate aqueous production in cases of PCD. Besides, silicone oil tamponade may not be needed, as posterior pole is already attached in those cases. Despite not using endoscopy, this approach led to good surgical outcomes, with long term visual and IOP stabilization.

## Electronic supplementary material

Below is the link to the electronic supplementary material.

Supplementary Material 1

## Data Availability

No datasets were generated or analysed during the current study.
